# Cumulative Signaling Through NOD-2 and TLR-4 Eliminates the *Mycobacterium Tuberculosis* Concealed Inside the Mesenchymal Stem Cells

**DOI:** 10.3389/fcimb.2021.669168

**Published:** 2021-07-07

**Authors:** Mohammad Aqdas, Sanpreet Singh, Mohammed Amir, Sudeep Kumar Maurya, Susanta Pahari, Javed Naim Agrewala

**Affiliations:** ^1^ Division of Cell Biology and Immunology, CSIR-Institute of Microbial Technology, Chandigarh, India; ^2^ Immunology Laboratory, Center for Biomedical Engineering, Indian Institute of Technology, Ropar, India

**Keywords:** tuberculosis, mesenchymal stem cell, NOD-2, TLR-4, autophagy

## Abstract

For a long time, tuberculosis (TB) has been inflicting mankind with the highest morbidity and mortality. Although the current treatment is extremely potent, a few bacilli can still hide inside the host mesenchymal stem cells (MSC). The functional capabilities of MSCs are known to be modulated by TLRs, NOD-2, and RIG-1 signaling. Therefore, we hypothesize that modulating the MSC activity through TLR-4 and NOD-2 can be an attractive immunotherapeutic strategy to eliminate the *Mtb* hiding inside these cells. In our current study, we observed that MSC stimulated through TLR-4 and NOD-2 (N2.T4) i) activated MSC and augmented the secretion of pro-inflammatory cytokines; ii) co-localized *Mtb* in the lysosomes; iii) induced autophagy; iv) enhanced NF-κB activity *via* p38 MAPK signaling pathway; and v) significantly reduced the intracellular survival of *Mtb* in the MSC. Overall, the results suggest that the triggering through N2.T4 can be a future method of immunotherapy to eliminate the *Mtb* concealed inside the MSC.

## Introduction

Tuberculosis (TB) is the cause of 2 million deaths each year and an estimated 1.8 billion people with latent disease worldwide (Mwaba et al., 2020). It is one of the top 10 diseases in terms of high morbidity and mortality worldwide (Herbert et al., 2014). Currently, drug-resistant TB is a major threat to mankind and quite common in TB endemic countries viz., India and China. Even though the available drugs remain the mainstay for the treatment of TB, certain limitations, such as their narrow therapeutic index and the associated toxicities, dilute their effectiveness (Forget and Menzies, 2006; Trauner et al., 2014). Due to its long duration, many patients fail to abide by the current regimen and quit before the completion of the course. This leads to the development of the very lethal drug-resistant TB (Munro et al., 2007). Innate and adaptive immune responses are responsible for protecting against invading pathogens. Early events include the engulfment of *Mtb* by the alveolar macrophages and dendritic cells, followed by their bactericidal mechanisms, such as the generation of reactive nitrogen intermediates (RNI) and reactive oxygen intermediates (ROI) (Sia and Rengarajan, 2019). Cytokines (IFN-γ, TNF-α, IL-6, IL-12, IL-17, and IL-23) and chemokines (CCL2, CCL3, CCL5, CXCL8, and CXCL10) help in restricting the *Mtb* burden and recruiting other immune cells to the site (Peters and Ernst, 2003; Saunders and Britton, 2007). *Mtb* has successfully evolved specialized immune evasion strategies that permit it to establish, multiply, and extend its infection within the host. Macrophages are the primary cells for *Mtb* infection. The bug acquires various strategies to persist in a dormant state in the hostile environment of the macrophage by inhibiting phagosome-lysosome (PL) fusion and de-acidification of lysosomes; thus, it averts its degradation and killing (Russell, 2013). Another mechanism of circumvention is the neutralization of reactive oxygen radicals by secretion of powerful anti-oxidants (Kumar et al., 2011). Most importantly, the lipid-rich cell wall of *Mtb* shields it from various defensive mechanisms. Thus, it becomes difficult to eliminate the latent form of *Mtb* with the current regimen and demands an urgent need for novel remedies for treating TB (Gomez and McKinney, 2004).

Recently, many studies have illustrated that bone marrow mesenchymal stem cells (MSCs) may provide a niche for shielding latent *Mtb*. MSCs are multipotent cells with a prospect to differentiate into adipocytes, osteocytes, chondrocytes, and neuronal cells. In the murine model of TB, rapid dissemination of *Mtb* was noticed, after aerosol exposure from the primary infection sites to bone marrow; where it infected MSCs (Garhyan et al., 2015). The murine model of TB dormancy demonstrates long-term intracellular viability and maintenance of *Mtb* in the MSCs (Das et al., 2013). It has been demonstrated that MSCs have a high number of ATP-binding cassette (ABC) transporter efflux pumps, which expel anti-TB drugs from host cells. Interestingly, viable *Mtb* is seen in the MSCs of patients, who had undergone successful anti-TB chemotherapy (Beamer et al., 2014). Thus, it can be inferred from these findings that *Mtb* can successfully hide in the MSCs until it gets ambient conditions to reactivate itself.

Innate immunity is the first line of defense, which subsequently imparts a significant impact on adaptive immune responses (Hoebe et al., 2004). Toll-like receptors (TLRs), NOD-like receptors (NOD-2), and RIG-like receptors (RIG-1) serve as a frontline defense system to defend against pathogens. These potentiate the ability of innate cells to recognize and subsequently respond to microbial infections (Kawai and Akira, 2011). Our group has already shown the importance of various innate signaling molecules against *Mtb* (Khan et al., 2016b; Khan et al., 2016c; Pahari et al., 2016; Pahari et al., 2020). MSCs substantially express an array of innate receptors like TLRs, NOD-2, or RIG-1 (Kim et al., 2010; Lei et al., 2011; Yang et al., 2013). TLRs are well-defined molecules that play an important role in the differentiation and self-renewal of MSC (Hwa Cho et al., 2006; Pevsner-Fischer et al., 2007). Recently, the switch of pro-inflammatory from anti-inflammatory polarization was also accredited to the activation of MSCs by delivering signals through TLRs (Waterman et al., 2010). This shows that the MSCs can be stimulated by signaling through TLRs, NLRs, and RIGs.

Based on the above-mentioned findings, the current study was designed to exploit the immunomodulatory potential of TLR-4 and NOD-2 in eliminating *Mtb* concealed inside the MSCs. Interestingly, signaling MSCs through NOD-2 and TLR-4 exhibited augmented secretion of pro-inflammatory cytokines, improved co-localization of *Mtb* in lysosomes, and significantly cleared the intracellularly masked mycobacterium. Mechanistically, stimulation of MSCs through NOD-2 and TLR-4 activated NF-κB activity *via* the p38 MAPK pathway and induced autophagy. In future, this novel strategy of host-directed therapy may open new avenues to eradicate *Mtb* hidden within the MSC.

## Materials and Methods

### Animals

Female BALB/c mice (6–8 weeks) were obtained from the Animal Facility, CSIR-Institute of Microbial Technology, Chandigarh, India. All the animal experiments were performed as approved by the ‘Institutional Animal Ethics Committee’ (IAEC) and accomplished according to the National Regulatory Guidelines issued by the ‘Committee for the Purpose of Control and Supervision of Experiments on Animals’ (No. 55/1999/CPCSEA), Ministry of Environment and Forest, Government of India.

### Strains of Mycobacterium

The *Mycobacterium tuberculosis (Mtb)* strains (H37Rv, H37Ra) were obtained from Dr. V. M. Katoch (National JALMA Institute for Leprosy and Other Mycobacterial Diseases, Agra, India). Mycobacterium strains were grown and cultivated in Middlebrook 7H9 broth supplemented with glycerol (0.2%), tween-80 (0.05%), dextrose, albumin, and catalase. Bacterial viability was enumerated through colony-forming units (CFUs) by plating them on Middlebrook 7H11 medium, supplemented with dextrose, albumin, oleic acid, and catalase after 21d of plating.

### Antibodies and Reagents

The standard reagents and chemicals were obtained from Sigma (St. Louis, MO). Recombinant cytokines and antibodies of IL-6, IL-12, TNF-α, and IL-10 were purchased from BD Biosciences (San Diego, CA). Fluorochrome-labelled antibodies (CD29 FITC, CD34 eFluor 660, CD44 PerCP-Cyanine5.5, CD45 APC, and Sca-1 PE) were purchased from eBiosciences (San Diego, CA) unless otherwise mentioned. Fetal bovine serum (FBS) was from GIBCO. LPS and N-glycolyl MDP used as ligands for TLR-4 and NOD-2 in the experiments were procured from InvivoGen (San Diego, CA). Oil Red-O stain was bought from Himedia (Mumbai, India). Alizarin Red S stain was acquired from Sigma (St. Louis, MO).

### Isolation of Bone-Marrow-Derived Mesenchymal Stem Cells From Mice

Mouse MSCs were isolated according to a protocol reported previously (Huang et al., 2015). Briefly, the tibia and femur bones were taken from the hind limb of BALB/c and kept in sterile phosphate buffer saline (PBS) (1X), after the removal of all residual soft tissues. Then, with the help of a 23G needle and syringe, bone cavities were flushed with Dulbecco’s modified Eagle’s medium (DMEM) having 10% heat-inactivated FBS, 1X penicillin-streptomycin, and 2 mM L-glutamine. Bone cavities were flushed repeatedly to obtain enough marrow cells. The cells were then cultured in cell culture dishes (100mm) for 5 days in 5% CO_2_/37°C. Later, the cells were washed twice with PBS (1X) and digested with trypsin (0.25%) containing ethylenediaminetetraacetic acid (EDTA) (0.02%) at RT for 2 min followed by trypsin neutralization with DMEM + FBS (10%). The cells were then centrifuged at 2000 rpm for 3 min and replated at a split ratio of 1:3 in fresh complete media. The experiments were performed when the MSCs showed a homogeneous pattern after several set of passages. The MSCs were harvested, washed, and cultured for the experiments.

The activation of MSC was done by signaling through NOD-2 and TLR-4. The MSCs (2x10^5^ cells/ml) were stimulated with a combination of N-glycolyl MDP (10 µg/ml) and ultra-purified LPS (5 ng/ml), the ligands of NOD-2 and TLR-4, respectively. The control cultures consist of unstimulated MSCs or cultured with either the ligand of NOD-2 or TLR-4. The cells were cultured in DMEM + FBS (10%) for 48h at 5% CO_2_/37°C. The culture supernatants (SNs) were harvested after 48h for the estimation of cytokines by ELISA and cells for the isolation of total RNA at 6h to perform RT-PCR.

### Estimation of Cytokine Secretion by ELISA

The cultures were set as mentioned above, and the cytokines (IL-12, IL-6, TNF-α, and IL-10) were estimated in the culture SNs of the MSCs by ELISA methods, according to the manufacturer’s instructions. Briefly, ELISA plates were coated with antibodies to mouse IL-12 (2μg/ml), IL-6 (2μg/ml), TNF-alpha (2µg/ml) or IL-10 (4μg/ml) in phosphate buffer (0.01 M Na_2_HPO_4_, pH 9.2, and pH 6, respectively) at 4°C for overnight. Blocking was performed with 1% BSA at RT for 2h. Later, SNs (50μl/well) were added in the wells, or their respective recombinant cytokines as standards, and kept at 4°C overnight. Then, the respective biotinylated anti-mouse IL-12 (2μg/ml), IL-6 (2μg/ml), TNF-alpha (2µg/ml) or IL-10 (2μg/ml) antibodies were added into plates and incubated for 2h at RT. Afterward, avidin-HRP (1:10,000) was added and incubated at 37°C for 45 min. After each incubation, regular steps of washing were carried out. Subsequently, the color was developed using H_2_O_2_-OPD substrate-chromogen, and the reaction was stopped by the addition of 7% H_2_SO_4_ in the plates. The plates were then read at 492 nm in an ELISA reader. Serial dilutions of recombinant cytokines (rIL-6, rIL-12 and rIL-10) were used to plot standard curves for the estimation of cytokines in SNs. Results of ELISA were expressed in pg/ml.

### RT-qPCR for the Quantification of *IL-12, IL-6, TNF-α, IL-10, iNOS*, and *TGF-β*

Isolation of total RNA was performed using TRIzol reagent from MSCs stimulated with N2.T4 (MSC^N2.T4^) for 6h, according to the manufacturer’s instruction (Invitrogen, Carlsbad, CA). Briefly, quantification of RNA was done using the NanoDrop spectrophotometer. The purity of all isolated RNA was in the range of 1.90 to 2.00 upon measured at A260/A280 (BioTek, Winooski, VT). The cDNA was synthesized using the Maxima first-strand cDNA synthesis kit for RT-qPCR (Thermo Fischer Scientific, K1642). Amplification-grade DNase1 (Sigma Aldrich, AMPD1-1KT) was used for removing DNA contamination from RNA samples. RNA samples (1μg) were treated with DNase1 (1U) in the reaction buffer for 15 min. DNase activity was stopped with the addition of a stop solution followed by incubating samples at 70°C for 10 min. Analysis was performed by the comparative Ct method, whereas normalization of the Ct values was done against a housekeeping control β-actin. Relative gene expression was determined using the comparative Ct method as 2(^-ΔΔ^Ct), where ΔCt = Ct (gene of interest) - Ct (normalizer = β-actin) and the ΔΔCt = ΔCt (sample) - ΔCt (calibrator). RT-qPCR, along with the analysis of data, was carried out using the ABI 7500 Fast Real-time PCR system (Applied Biosystems, Chromas, Singapore). Results were presented as a relative expression (fold change). The primer sequences for RT-qPCR are mentioned below.

TNF-α

Fwd 5’-CCTGTAGCCCACGTCGTAG -3’

Rev 5’-GGGAGTAGACAAGGTACAACCC -3’

TGF-β

Fwd 5’- TGACGTCACTGGAGTTGTACGG-3’

Rev 5’-GGTTCATGTCATGGATGGTGC-3’

β-actin

Fwd 5’-AGAGGGAAATCGTGCGTGAC-3’

Rev 5’-CAATAGTGATGACCTGGCCGT-3’

IL-6

Fwd 5’-GAGGATACCACTCCCAACAGACC-3’

Rev 5’-AAGTGCATCATCATCGTTGTTCATACA-3’

IL-12

Fwd 5’-GGAAGCACGGCAGCAGCAGAATA-3’

Rev 5’-AACTTGAGGGAGAAGTAGGAATGG-3’

iNOS

Fwd 5’-AACGGAGAACGTTGGATTTG-3’

Rev 5’-CAGCACAAGGGGTTTTCTT-3’

### The Characterization of Phenotypic Markers of MSCs

For the immunophenotype assay, flow cytometry analysis was carried out. MSCs (2X10^5^ cells) were washed and harvested from the plates after they attained the confluency. Cells were then incubated with Fc receptor blocking antibody (anti-CD16/32) for 20 min at 4°C. Subsequently, cells were stained with fluorochrome-conjugated Abs specific for CD34, CD44 (osteopontin and hyaluronate marker), Sca-1 (stem cell antigen-1), CD29 (Integrin β-1), and CD45 (pan-leukocyte marker) Abs at 4°C for 30 min. Washing was done at each step. Later, cells were fixed using paraformaldehyde (1X) and acquired on the FACS ARIA flowcytometer. The data were analyzed using BD DIVA software (BD Biosciences, San Jose, CA).

### Evaluation of the Differentiation of MSCs

For adipogenesis differentiation assay, MSCs (2 X 10^5^/well) were seeded in a 6-well plate in DMEM+10% FBS complete media. The next day, a fresh medium was poured along with a pre-warmed complete adipogenesis differentiation medium (StemPro^®^ Adipogenesis Differentiation Kit; A10070-01) and kept at 37°C in a 5% CO_2_ incubator. Cultures were fed every 3–4 days with adipogenesis differentiation media. After 21 days, cells were washed twice with PBS (1X) followed by fixation with 10% formalin. Cells were then stained with Oil red O (Himedia: TC256) stain. The pictures were taken under a phase-contrast microscope (10X) (Olympus IX71, Tokyo, Japan).

For osteogenesis differentiation assay, MSCs (2 X 10^5^/well) were seeded in a 6-well plate in DMEM+10% FBS complete media. After 24h, fresh media (DMEM+10% FBS) was added along with a pre-warmed osteogenesis differentiation medium (StemPro^®^ Osteogenesis Differentiation Kit; A10072-01). Cultures were replenished every 3–4 days with osteogenesis differentiation media. After 3 weeks, fixation of cells was done using formalin (10%) and stained for calcium deposition with Alizarin Red S (Sigma Aldrich, St. Louis, MO).

### *In Vitro* Infection of MSCs With *Mtb* and Determination of CFUs

*Mtb* (H37Rv) was grown till the mid-log phase and stored in a glycerol stock at -80°C. Later, the bacterium was thawed, and MSCs (2 X 10^5^/well) were infected with *Mtb* at multiplicity of infection (MOI) 1:5 for 4h. The extracellular bacteria were eliminated by treatment with gentamicin (10 μg/ml) for 1h and then washed with PBS (1X). *Mtb*-infected MSCs were then stimulated with ligands of NOD-2 and TLR-4 (N2.T4) for 48h and plates were kept in a CO_2_ (5%) incubator at 37°C. Later, the SNs were collected for the cytokine ELISA and cells were lysed with saponin (0.1%). 100X serial dilutions of cell lysate were plated on 7H11 agar plates. Plates were kept in an incubator at 37°C. Bacterial colonies were enumerated for CFUs after 3 weeks.

### Tracking of *Mtb* Into Autolysosomes by LysoTracker Red Staining

MSC (2 X 10^5^ cells/well) were infected with GFP-*Mtb* (H37Ra) for 4h at MOI of 1:5. The extensive washing was done with PBS (1X) to get rid of extracellular *Mtb* followed by gentamicin (10 µg/ml) treatment for 1h. The cells were stimulated with N2.T4 for 12 h. The cells were stained with 200 nM of LysoTracker Red (prepared in media) for 20 min at 37°C/5% CO_2_. The cells were then washed twice with PBS (1X) followed by fixing with PFA (4%). After fixation, the nucleus was stained with DAPI (1 µg/ml) for 10 min, followed by washing three times with PBS (1X). The coverslips were mounted onto the slide with help of a mounting reagent and observed under a confocal microscope (Nikon A1R, Nikon, Yokohama, Japan), using the lasers 488 nm (GFP-*Mtb*H37Rv), 561 nm (LysoTracker Red), and 405 nm (DAPI) with the same power set for controls. In total, 10 random fields were imaged, and the percentage of *Mtb* containing autophagosomes colocalized with lysosomes was counted.

### Evaluation of Signaling in MSC^N2.T4^ by Western Blotting

MSC (2 X 10^6^ cells/well) were stimulated with N2.T4 (MSC^N2.T4^) for 24h. Cells were then harvested, washed, and lysed in a lysis buffer (RIPA buffer, protease and phosphatase inhibitor cocktail). Proteins in the lysate were then estimated and equal concentrations of lysates were subjected to SDS-PAGE electrophoresis. After transfer to the nitrocellulose membrane, followed by blocking with BSA (2%), the membranes were then immunoblotted with Abs specific for LC3-I/LC3II, beclin-1, phospho-p38/p38, and NF-κB-p65. Actin was used as a loading control. The blots were developed using a chemiluminescence kit (Amersham Pharmacia Biotech, Buckinghamshire, UK). Blots were then scanned with ImageQuant LAS 4000 (GE Healthcare, Pittsburgh, PA). The image analysis was performed with ImageJ software.

### Statistical Analysis

All data were analyzed using student “t-test” and one-way analysis of variance (ANOVA) with post-Tukey-Kramer multiple comparisons test by Graph Pad Prism 6 software (GraphPad Software, La Jolla, CA). Data were expressed as mean ± SD. The *p<0.05 was considered significant.

## Results

### Characterization of MSCs Isolated From the Bone Marrow

**Figure 1:**
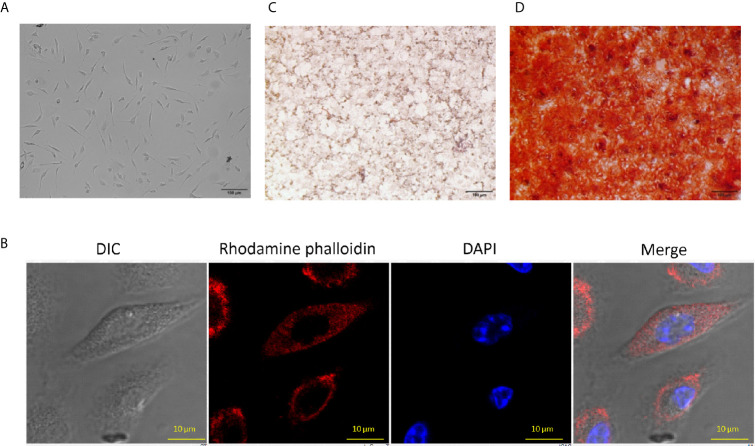
Characteristics of mouse bone marrow-derived mesenchymal stem cells (MSC). **(A)** MSCs were isolated from mouse bone marrow and expanded to several passages that showed fibroblast-like morphology and arranged in the swirl. The photographs were taken using an Olympus IX71phase contrast microscope (10X). **(B)** MSCs were stained with rhodamine phalloidin (red) and DAPI (blue) and observed under a confocal microscope (60X). **(C)** Intercellular lipid vacuoles were stained with Oil Red O to determine the adipogenic differentiation potential of MSC. The pictures were taken using an Olympus phase contrast microscope (10X). **(D)** The MSC were stained with the Alizarin Red S to assess the osteogenic differentiation by the formation of calcium nodules. The pictures were clicked using a phase contrast microscope (10X). The photographs shown are the representative of three experiments.

Bone-marrow-derived MSCs were isolated and cultured, as described elsewhere (Huang et al., 2015). The cellular morphology of MSCs was spindle shaped (fibroblast like), as observed under a microscope (**Figures 1A, B**). Further, isolated MSCs were stained with rhodamine phalloidin (selectively binds to F-actin) and DAPI to study their morphological features. MSCs have the potential to differentiate into various lineages (Robert et al., 2020). Subsequently, the MSC were checked for their adipogenic and osteogenic differentiation characteristics. We observed red-colored intracellular lipid vacuoles after Oil Red O staining (**Figure 1C**). Further, Alizarin Red S stained the calcium nodules deposition, which confirmed the osteoblasts formation in MSC (**Figure 1D**). It has been reported that the MSCs can be phenotypically characterized by the expression of several positive and negative markers on their surface (Soleimani and Nadri, 2009). Flow cytometric analysis confirmed the expression of Sca-1, CD44, and CD29 markers and the absence of CD45 molecules (**Supplementary Figure 1**). Thus, the cells isolated from bone marrow were phenotypically and functionally characterized as MSCs and all the subsequent experiments were performed using these cells.

### Signaling Delivered Through NOD-2 and TLR-4 Stimulates MSCs

**Figure 2:**
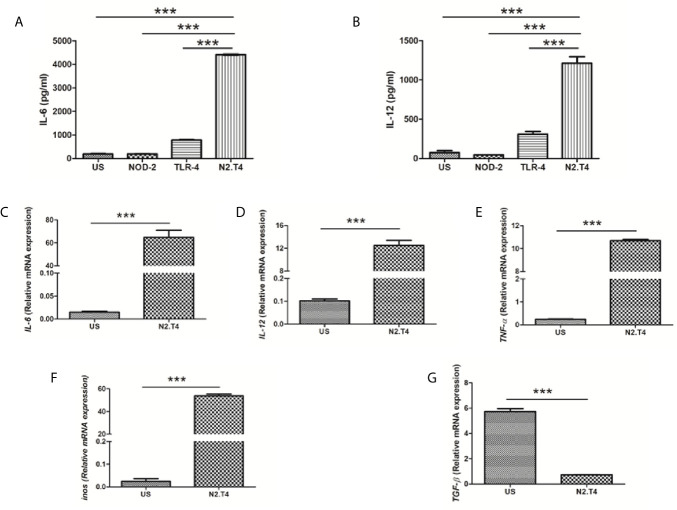
Cumulative signaling through NOD-2 and TLR-4 stimulates MSC to release IL-6 and IL-12. **(A, B)** MSCs were cultured with the ligands of NOD-2 and TLR-2 (N2.T4). The controls were set using unstimulated MSCs (US) or stimulated with either the ligand of NOD-2 (NOD-2L) or TLR-4 (TLR-4L). The culture SNs were collected after 48h and estimated for the production of **(A)** IL-6 and **(B)** IL-12 cytokines by ELISA. **(C–G)** The MSCs were stimulated as indicated above **(A, B)** and the mRNA expression of *Il6* **(C)**, *Il12* **(D)**, *Tnfa* **(E)**, *Inos* **(F)** and *Tgfb* **(G)** was performed by RT-PCR. Graphs depict the ‘mRNA expression relative to unstimulated (US) control’. Data expressed as mean ± SD are representative of two independent experiments. Statistical analysis was done using one way ANOVA for ELISA and unpaired t-test for RT-PCR. ***p < 0.001.

MSC expresses innate receptors, such as TLRs, NOD-2, and RIG-1 (Kim et al., 2010; Lei et al., 2011; Yang et al., 2013). Hence, we thought whether ligation of NOD-2 and TLR-4 can stimulate MSCs. Unfortunately, we could not observe any statistical change in the activation of MSCs, as depicted by the release of IL-6 and IL-12 (**Figure 2A**). Intriguingly, when we activated MSCs by combinatorial signaling through NOD-2 and TLR-4 (MSC^N2.T4^), a substantial (p<0.001) increase in the release of IL-6 and IL-12 was noted, as compared to the control MSCs stimulated with the ligand of either NOD-2 or TLR-4 (**Figures 2A, B**). Therefore, the combination of ligands of NOD-2 and TLR-4 (N2.T4) were used in all the subsequent experiments to stimulate MSCs (MSC^N2.T4^). It has been reported that TNF-α, IL-12, IL-6, and iNOS play a crucial role in curbing the intracellular growth of pathogens like *Mtb*, leishmania, salmonella, HIV, etc. (Black et al., 1990; Jankovic et al., 2002; Chakravortty and Hensel, 2003). Further, this observation was corroborated through gene expression in MSCs by RT-qPCR. Besides *IL-12* (p<0.001) and *IL-6* (p<0.001), *iNOS* (p<0.001) along with *TNF-α* (p<0.001) also showed elevation in their expressions upon stimulation with N2.T4 (**Figures 2C–F**). Contrary to this, there was a substantial (p<0.001) reduction in the anti-inflammatory cytokine TGF-β (**Figure 2G**). Hence, we observed a shift from anti-inflammatory to pro-inflammatory phenotype after triggering MSCs through N2.T4.

### Signaling *Mtb*-Infected MSCs Through N2.T4 Augments the Release of Pro-Inflammatory Cytokines and Constrains the Intracellular Growth of the *Mtb*

**Figure 3:**
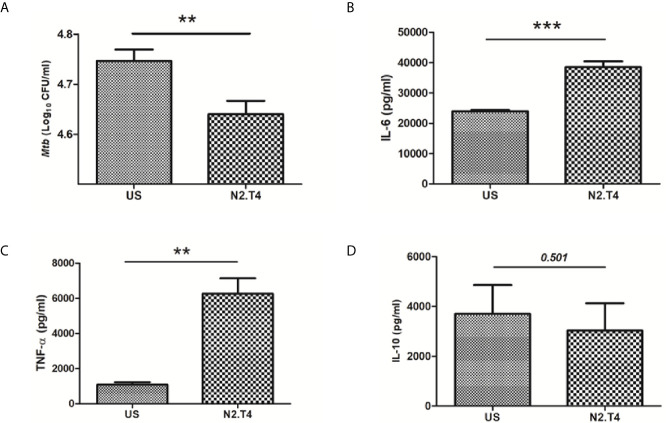
Signaling MSCs through N2.T4 restricted the intracellular growth of *Mtb* and elicited the secretion of pro-inflammatory cytokines. **(A)** MSCs were infected with *Mtb* (GFP-H37Ra) for 4h and then stimulated with N2.T4 for 48h. Later, cells were lysed and CFUs were enumerated on 21d by CFU assay. The Bar diagram represents the mean ± SEM and is indicative of three independent experiments. Statistical analysis was done using one-way ANOVA. The culture SNs were collected after 48h for determining the yield of cytokines **(B)** IL-6, **(C)** TNF-α and **(D)** IL-10 by ELISA. Statistical analysis was done using an unpaired t-test. The inset represents p value. **p < 0.01 and ***p < 0.001.

Recent studies have shown that *Mtb* can successfully infect and hide inside the MSCs (Das et al., 2013). Hence, we were curious to monitor the influence of N2.T4 signaling of MSCs (MSC^N2.T4^) on the intracellular survival of the bacterium. Interestingly, MSCs infected with *Mtb* (H37Rv) upon N2.T4 stimulation significantly (p<0.01) restricted the bacterial burden compared to unstimulated (US) MSCs (**Figure 3A**). Furthermore, remarkable elevation was noticed in the secretion of pro-inflammatory cytokines TNF-α (p<0.01) and IL-6 (p<0.001) by MSC^N2.T4^ (**Figures 3B, C**). The non-significant decrease was observed in the level of anti-inflammatory cytokine IL-10 (**Figure 3D**). These results suggest that combinatorial stimulation of MSCs through NOD-2 and TLR-4 can successfully restrict the intracellular growth of *Mtb* masked inside these cells.

### Signaling of MSC^N2.T4^ Augments the Co-Localization of *Mtb* in Lysosomes

**Figure 4:**
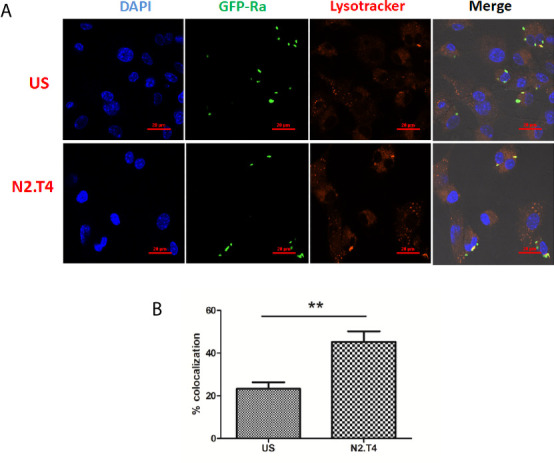
Triggering MSCs through N2.T4 induced trafficking of *Mtb* to autolysosomes. **(A)** MSCs (2 X 10^5^ cells/well)were infected with GFP-*Mtb* (H37Ra) for 4h followed by signaling through N2.T4 for 12h. Later, the lysosomes were stained with LysoTracker Red for 20 min. The cells were fixed with PFA (4%) and slides were prepared for confocal microscopy. The panel shows the representative image of *Mtb* (green channel, 488 nm) colocalization with LysoTracker Red (red channel, 561 nm) (60X). **(B)** The data from **(A)** are also illustrated as a graph depicting the percentage of colocalization of *Mtb* with the lysosome. The data shown as mean ± SEM are representative of three independent experiments. Statistical significance was determined using the unpaired t-test. **p < 0.01.

One of the potent mechanisms responsible for the killing of various intracellular pathogens is the lysosomal degradation pathway (Jo, 2010). Scavenger receptors like MARCO and SR-B1 present on MSCs play an important role in internalizing *Mtb* (Khan et al., 2017). Phagosome lysosome fusion is decisive for the eradication of the intracellularly hidden *Mtb* (Khan et al., 2017). However, *Mtb* has a unique tendency to evade the immune system by inhibiting the phagosome lysosome fusion; thereby can successfully survive in the hostile environment of macrophages and MSC (Jamwal et al., 2016). Consequently, we next studied the signaling of MSCs through N2.T4 and its influence on the intracellular trafficking of *Mtb*. MSCs were infected with *Mtb* overexpressing GFP. Further, MSCs were stained with LysoTracker Red dye to monitor the acidification of the lysosome. Later, the signaling was delivered in MSCs through N2.T4. We observed that *Mtb* inhibited the phagosome lysosome fusion of the infected MSC (**Figure 4A**). It was interesting to note that MSC^N2.T4^ could efficiently overcome the *Mtb* induced inhibition of phagosome lysosome fusion, as demonstrated by a significant (p<0.01) increase in the co-localization of *Mtb* and LysoTracker Red dye (**Figures 4A, B**). These results signify that the mechanism responsible for the killing of *Mtb* by MSC^N2.T4^ may be operating through the enhanced fusion of phagolysosomes (**Figures 3A** and **4A, B**). Hence, N2.T4 may have an important immunotherapeutic role in eliminating *Mtb* concealed in the MSCs.

### Signaling Through N2.T4 Induces Autophagy in MSC

**Figure 5:**
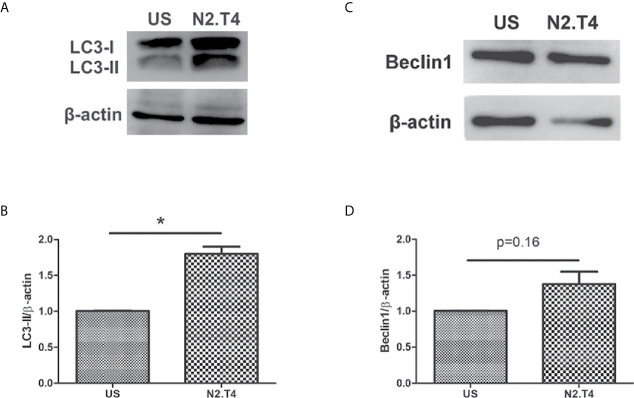
N2.T4 signaling induced autophagy in MSC. MSC (2 X 10^6^ cells/well) were stimulated with either N2.T4 or left untreated for 24h. The cells were lysed with a RIPA buffer containing a protease inhibitor cocktail. The samples were subjected to SDS-PAGE and Western blotting. The blots were probed with antibodies against autophagy markers LC3 and beclin-1 **(A, C)**. The densitometric analysis of LC3 and beclin-1, normalized with β-actin is represented in graphs **(B, D)**, respectively. The data shown are representative of three independent experiments. The inset represents p value. *p < 0.05.

The intrinsic autophagy mechanism is known to inhibit the growth of *Mtb* inside MSCs (Khan et al., 2017). The transition of LC3-I to LC3-II is evidence of autophagy. Consequently, we next checked the induction of autophagy in MSCs^N2.T4^. Interestingly, we observed conversion of LC3-I to LC3-II (**Figures 5A, B**). These results suggest a novel role of signaling of MSC through N2.T4 in inducing autophagy. Beclin-1 is a well-known initiator and master regulator of autophagy. Thus, the levels of beclin-1 in MSC^N2.T4^ were monitored. We observed an increased level of beclin-1 in MSC^N2.T4^ (**Figures 5C, D**). The modulation of the expression of markers LC3 and beclin-1, suggests the role of autophagy as a possible mechanism operating in curtailing the intracellular growth of *Mtb* in MSC^N2.T4^.

### N2.T4 Signaling of MSC Induces NF-κB Activity *via* p38 MAPK Pathway

**Figure 6:**
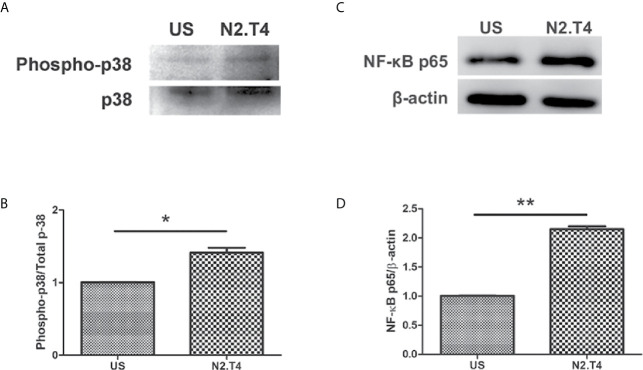
Stimulation of MSCs through N2.T4 induced NF-κB activity *via the* p38 MAPK pathway. MSCs were stimulated with the ligands of N2.T4 for 24 hours and control cells were left unstimulated (US). The cells were lysed with a RIPA buffer containing protease inhibitor cocktail. Total cell lysates were subjected to SDS-PAGE and Western blotting. The blots were probed with antibodies against **(A)** phosphor-p38/p38 and **(C)** NF-κB-p65 with densitometric analysis normalized with β-actin, represented in graphs **(B, D)**, respectively. The data shown are representative of two independent experiments. *p < 0.05, **p < 0.01.

The TLR signaling is known to initiate MAPK pathways through MyD88 molecules (Kim et al., 2010). Further, NF-κB is activated after degradation of IκB and translocates into the nucleus to affect the target genes, denoting the activation status of the cell. We noticed a substantial induction of p38 in MSC^N2.T4^, as compared to unstimulated cells (**Figures 6A, B**). Furthermore, we noticed increased levels of NF-κB-p65 (**Figures 6C, D**). These results suggest the importance of combinatorial signaling of NOD-2 and TLR-4 in enhancing the activation and functionality of the MSC^N2.T4^ to kill covert *Mtb*.

## Discussion

*Mycobacterium tuberculosis (Mtb)* is one of the most astute pathogens that the human race has ever encountered. This is evident by the fact that it understands the mechanism of i) living in a dormant state in a hostile environment of the host; ii) impairing the functioning of BCG vaccine; iii) developing resistance against the drugs designed to kill it; iv) developing coalition with HIV; and v) attacking malnourished individuals. Further, the problem has been compounded with a recent discovery of enduring residency of *Mtb* in the multipotent cells i.e. MSC (Das et al., 2013; Beamer et al., 2014). Consequently, *Mtb* continues to make its elimination a daunting task for the scientific community.

Only 5–15% of people infected with *Mtb* develop TB. This indicates 85–95% of infected individuals develop a remarkably strong immunity to remain protected throughout their lives. This indicates that host immunity plays a primary role in protecting against TB. Therefore, boosting host immunity can play a cardinal role in protecting against *Mtb*. Recently, host-directed therapy (HDT) has gained considerable momentum following the observation that it not only controls the infection and devastating inflammatory responses inflicted by *Mtb* to the host but also the emergence of drug-resistant strains of the bacterium (Zumla et al., 2016; Young et al., 2020). Exploration and exploitation of the molecules of innate immunity may be an estimable idea for bolstering host immunity since innate immunity plays an imperative role against *Mtb* (Fremond et al., 2004). The NOD-2 is an important receptor of innate immunity because its role has been reported in effectively modulating the cell’s immunity (Divangahi et al., 2008; Jo, 2008). Likewise, TLR-4 plays a crucial function in boosting immunity against many pathogens (Kleinnijenhuis et al., 2011; Mortaz et al., 2015). Recently, we have demonstrated a combinatorial role of NOD-2 and TLR-4 in substantially augmenting the functionality of dendritic cells in priming T cells and killing *Mtb* (Khan et al., 2016a). Mesenchymal stem cells express various innate receptors on their surface, including NOD-2 and TLR-4. These receptors assist in the recognition and delivering signals on encounters with the pathogens (Kim et al., 2010; Lei et al., 2011; Yang et al., 2013). Signaling through innate receptors can polarize MSCs from anti-inflammatory to pro-inflammatory phenotype (Waterman et al., 2010).

Based on the above-mentioned studies, we thought to examine the influence of signaling through NOD-2 and TLR-4 in modulating the activity of MSCs against *Mtb*. MSCs were infected with *Mtb*, and signaling was delivered using the ligands of NOD-2 and TLR-4. Following major findings emerged out of this study. MSC^N2.T4^ exhibited i) activation phenotype, as illustrated by the enhanced release of IL-6, IL-12, TNF-alpha, iNOS and decrease in TGF-β; ii) increased co-localization of *Mtb* in lysosomes; iii) induction of NF-κB activity *via* the p38 MAPK pathway; iv) augmented autophagy; and v) a decline in the survival of *Mtb* inside the MSCs.

NODs and TLRs are innately expressed on MSCs (Delarosa et al., 2012). NOD-2 and TLR-4 coordinate with each other in imparting protection against pathogens like *Mtb* (Khan et al., 2016c). Likewise, our study demonstrated that combinatorial signaling of TLR-4 and NOD-2 can activate MSCs and thereby can restrict the intracellular growth of *Mtb*. N-glycolyl MDP was selected as a NOD-2 agonist because it is 10–100 fold more effective, as compared to N-acetylated MDP (Coulombe et al., 2009). Ultrapure LPS was used for triggering TLR-4, which has remarkable adjuvant properties. Further, the Food and Drug Administration (FDA) has approved the immunotherapeutic use of the ligand of TLR-4 (Bohannon et al., 2013; Needham et al., 2013).

It was intriguing to note that the signaling of MSCs through NOD-2 and TLR-4 (MSC^N2.T4^) exhibited anti-*Mtb* immunity, as evidenced by a significant increase in the pro-inflammatory molecules IL-12, IL-6, TNF-α, and iNOS and reduction in anti-inflammatory cytokine TGF-β. This was further reflected by a substantial decline in the survival of *Mtb* in the MSCs. Mesenchymal stem cells express TLR-4 as a type I transmembrane glycoprotein (Takeda and Akira, 2005). The activation of TLR-4 requires adaptors and co-receptors (MD2, LMP, and CD14) for dimerization that facilitates MyD88/TRIF-dependent activation of the transcription factors (Zhu et al., 2006; Najar et al., 2017). It has been shown that the effect of LPS is compromised in MSCs derived from MyD88^−/−^ mice (Chu et al., 2019). Moreover, LPS has been shown to convert MSCs from anti-inflammatory to pro-inflammatory phenotype (Waterman et al., 2010). Further, TLR-4 activation enhanced the proliferation of MSCs (Pevsner-Fischer et al., 2007). Thus, indicating the potential role of TLR-4 in the signaling of MSCs.

In general, TLR stimulation activates MyD88-dependent and independent signaling pathways. MyD88 recruitment to TLRs triggers numerous signaling pathways *via* IRAKs, which subsequently initiate MAPK pathways. Therefore, to decipher the mechanism operating in restricting the survival of *Mtb* in MSC, we checked the level of the p38 MAPK signaling pathway. We observed elevated expression of p38 molecule in MSC^N2.T4^. It is already reported that the p38 phosphorylation activates NF-κB followed by subsequent translocation to the nucleus (Olson et al., 2007; Karunakaran and Ravindranath, 2009). Increased levels of NF-κB p65 in MSC^N2.T4^ were observed, as compared to unstimulated cells. Furthermore, it was observed that the decline in the survival of *Mtb* was through autophagy, as revealed by the modulation in the expression LC3 and beclin-1. Autophagy is an inherent quality of many stem cell types and is considered to be vital for their pluripotency, differentiation, and self-renewal (Phadwal et al., 2013). Autophagy has been described to exhibit a dual role in *Mtb* protection. Firstly, it targets the antigen for lysosomal degradation, and, secondly, it prevents the inflammatory reaction; thus protecting from tissue necrosis along with the associated pathology (Castillo et al., 2012).

## Conclusion

Overall, our results suggest that host-directed therapy through N2.T4 may be a good option for priming MSC to kill non-replicating quiescent intracellular *Mtb*. Further, the results suggest that this strategy may have enough potential in inhibiting the intracellular growth of drug-resistant *Mtb*. Finally, in future, this novel immunotherapeutic strategy may sufficiently contribute in successfully treating TB patients.

## Data Availability Statement

The original contributions presented in the study are included in the article/**Supplementary Material**. Further inquiries can be directed to the corresponding author.

## Ethics Statement

The animal study was reviewed and approved by Institutional Animal Ethics Committees (IAEC) of IMTECH, Chandigarh.

## Author Contributions

The concept, experiment designing, and data analysis were done by JA and MAq. The experiments were conducted by MAq, SS, SP, MA, and SM. The manuscript was written by JA, MAq, and SS. All authors contributed to the article and approved the submitted version.

## Funding

The work was carried out under funding support from the Council of Scientific and Industrial Research (CSIR), India. MAq received a fellowship from the Department of Science and Technology (DST), SS from the Indian Council of Medical Research (ICMR), MA from University Grant Commission, and SM and SP were the recipients of fellowships from CSIR.

## Conflict of Interest

The authors declare that the research was conducted in the absence of any commercial or financial relationships that could be construed as a potential conflict of interest.

## Supplementary Material

Supplementary Figure 1 The MSC were stained with the fluorochrome-labeled respective Abs and analyzed by flowcytometry for the expression of phenotypic markers viz CD44, CD29, Sca-1, and CD45. The black and red histograms represent unstained and stained MSCs, respectively.
