# Supplementary figures and images for: Cumulative Signaling Through NOD-2 and TLR-4 Eliminates the Mycobacterium Tuberculosis Concealed Inside the Mesenchymal Stem Cells
Source: Front Cell Infect Microbiol. 2021 Jul 7;11:669168. doi: 10.3389/fcimb.2021.669168 (PMC8294323; doi:10.3389/fcimb.2021.669168)

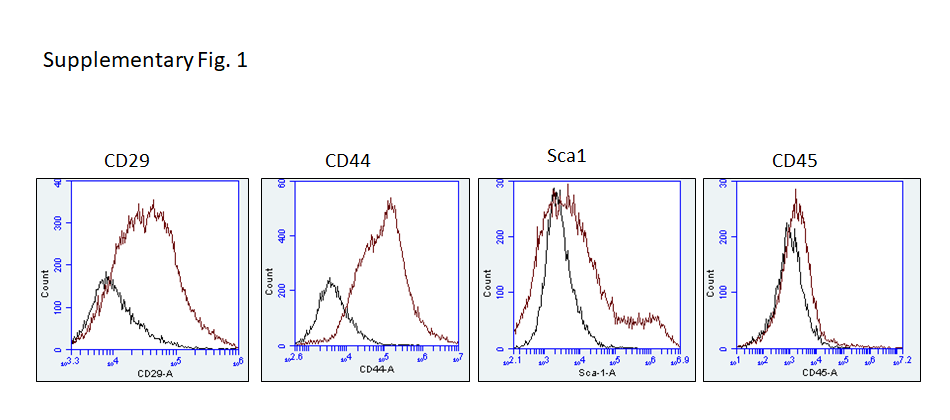

Supplement: Supplementary Figure 1 — The MSC were stained with the fluorochrome-labeled respective Abs and analyzed by flowcytometry for the expression of phenotypic markers viz CD44, CD29, Sca-1, and CD45. The black and red histograms represent unstained and stained MSCs, respectively. [file Image_1.tif]
